# Association of Depressive and Negative Symptoms with Quality of Life in Schizophrenia: A Cross-Sectional Inpatient Study

**DOI:** 10.3390/jcm15093442

**Published:** 2026-04-30

**Authors:** Jonas Montvidas, Paulina Petraitytė, Edas Kačerginskis, Algirdas Musneckis, Sonia Dollfus, Virginija Adomaitienė

**Affiliations:** 1 Department of Psychiatry, Lithuanian University of Health Sciences, LT-44307 Kaunas, Lithuania; paulina.petraityte@stud.lsmu.lt (P.P.); edas.kacerginskis@stud.lsmu.lt (E.K.); algirdas.musneckis@lsmu.lt (A.M.); virginija.adomaitiene@lsmu.lt (V.A.); 2 Department of Psychiatry, University of Cean Normandy, 14000 Caen, France; dollfus@cyceron.fr

**Keywords:** schizophrenia, quality of life, negative symptoms, depressive symptoms, primary negative symptoms, prominent negative symptoms

## Abstract

**Background/Objectives**: Identifying the factor most strongly associated with patients’ quality of life (QoL) is crucial for establishing treatment goals focused on improved recovery. This study aimed to determine whether sociodemographic factors, negative, or depressive symptoms have the strongest association with QoL. **Methods**: Inpatients diagnosed with schizophrenia were recruited. We collected data on sociodemographic factors, asked patients to rate their well-being on a subjective well-being scale, and evaluated their psychopathology using observer-rated psychometric scales (Positive and Negative Symptoms Scale (PANSS), Brief Negative Symptoms Scale (BNSS), and Calgary Depression Scale for Schizophrenia (CDSS) as well as self-rated scales (Self-evaluation Negative Symptoms Scale (SNS). QoL was evaluated using Short-Form 36 (SF-36). Patients were also divided into primary, prominent, and predominant negative symptom groups. We conducted correlation and linear regression analyses to identify which factors were most strongly associated with QoL. **Results**: In this study, 323 participants were included. The CDSS total score showed the strongest correlation with QoL scores, followed by negative symptoms assessed with the SNS. Positive and negative symptoms, assessed using either the PANSS or the BNSS, showed weak or insignificant correlations with QoL. Among sociodemographic factors, the subjective well-being score, previous history of hospitalization, or suicide attempts had the strongest correlation with QoL. CDSS scores were the variable with the strongest independent association with QoL in regression analysis. **Conclusions**: Depressive symptom severity showed the strongest and most consistent association with QoL across both correlation and multivariable analyses. These findings are hypothesis-generating and require longitudinal confirmation.

## 1. Introduction

The link between quality of life (QoL) and disease burden in patients with schizophrenia has been consistently demonstrated in research. According to a 2019 Global Burden of Disease study, schizophrenia was the 20th leading cause of disability worldwide, responsible for almost 2% of total years lived with disability [1]. This translates to a major economic burden of over 340 billion USD in the USA alone [2]. According to He et al., an improvement in QoL leads to better functioning and reduced disease burden [3]. Unfortunately, some studies suggest that symptom remission does not necessarily lead to a better QoL [4]. Moreover, according to Rhee et al., improving patients’ QoL greatly improves their caregivers’ QoL and functioning [5]. However, addressing predictors of quality of life can significantly reduce healthcare costs—sometimes by up to 49% [1,6,7]. Therefore, QoL is increasingly recognized as a key marker of recovery in schizophrenia. 

Understanding which symptoms have the greatest negative impact on QoL is paramount for setting treatment targets that promote better recovery, rather than simply reducing symptoms. Some studies find that every schizophrenia symptom group is inversely correlated with QoL variables [8]; other studies find that while some psychopathology domains, such as depressive and negative symptoms, have a stronger negative correlation with QoL, other symptom domains, such as positive symptoms, are not as strongly negatively correlated with QoL [9]. This creates a research gap, as it remains unclear which psychopathological domain should be the main focus for improving QoL. This may partly reflect the use of older or non-specific scales with known content validity limitations for negative and depressive symptom assessment, which might cause discrepancies in the results. Also, methodological choices, such as sample sizes and QoL scales, may explain these differences. Therefore, studies employing newer psychometric scales for symptom detection and further investigating the association between schizophrenia psychopathology and QoL are needed.

Moreover, negative symptoms can be grouped into primary, secondary, prominent, and predominant categories, and only a handful of papers report on the relationships among these types of negative symptoms and QoL. Recently, the European Medicines Agency (EMA) and the Food and Drug Administration (FDA) recommended that research on treatment options for negative symptoms should exclude secondary negative symptoms and include prominent or predominant negative symptoms [10,11,12,13]. Therefore, understanding the relationship between QoL and these different groups of negative symptoms has become even more important. However, it is important to note that neither EMA nor FDA has set clear criteria for any of these negative symptom groups.

Sociodemographic variables also influence the QoL of patients with schizophrenia. Multiple studies have examined the correlation between sociodemographic and QoL variables, yielding mixed results [4,14]. Some authors have reported that QoL is a gender-specific construct, with females having lower QoL [15]. However, it remains unclear to what extent these results can be replicated across all QoL domains. Other studies have found that age and illness duration are among the main factors contributing to poor QoL [4]. However, the results remain inconclusive. Finally, the hierarchy of influence among sociodemographic factors and psychopathological symptoms on QoL remains unclear.

Our primary aim is to examine whether sociodemographic factors, depressive symptoms, or negative symptoms are most strongly associated with QoL. We hypothesized that negative symptoms would show the strongest association.

## 2. Materials and Methods

This study is a part of a larger research project investigating the prevalence of negative symptoms and validating tools for assessing the psychopathology of schizophrenia among patients with schizophrenia in Lithuania [16].

This was a cross-sectional study; participants were recruited in an inpatient setting between January 2024 and August 2025. Inclusion criteria included diagnosis of a schizophrenia spectrum disorder according to the ICD-10 (F20–F29), aged between 18 and 65, and agreed to join the trial by signing the informed consent form. Exclusion criteria included comorbidity with intellectual disability, active substance abuse disorders, and/or severe somatic diseases. The sample size was calculated using Cochran’s formula for proportions with finite population correction. The population size was obtained from the public Lithuanian registry of disease prevalence: 22,777 patients were diagnosed with F20-F29 in Lithuania in 2024. Because the sample size was calculated for a study assessing the prevalence of negative symptoms, an expected prevalence of 70% was used. Applying finite population correction (population 22,777; expected prevalence 70%; 95% CI; 5% margin of error) the minimum required sample size was 318.

We collected sociodemographic data, including sex, age, weight, years of education, work capacity (according to the Lithuanian official grading system), and years of illness. We also asked participants whether they had attempted suicide or been previously hospitalized in a psychiatric ward. Finally, we asked each participant to rate their overall well-being on a Likert scale from 1 (very bad) to 10 (excellent). We called this rating the subjective well-being score. The severity of negative symptoms was assessed using the Positive and Negative Symptoms Scale (PANSS) negative subscale (PANSS NS), the Brief Negative Symptoms Scale (BNSS), and the Self-evaluation of Negative Symptoms (SNSs). Positive symptoms were evaluated using the PANSS positive subscale (PANSS PS).

The PANSS is a semi-structured interview with 30 items, each rated from one (absent) to seven (extreme). The PANSS has three subscores: positive symptoms (items P1–P7), negative symptoms (items N1–N7), and general psychopathology (items G1–G16) [17]. The Marder Negative Factor (MARDER NF) was calculated by summing items N1 (Blunted Affect), N2 (Emotional Withdrawal), N3 (Poor Rapport), N4 (Passive Social Withdrawal), N6 (Lack of Spontaneity in Conversation), and G7 (Motor Retardation) [18,19].

Depressive symptoms in schizophrenia were assessed using the Calgary Depression Scale for Schizophrenia (CDSS), a semi-structured 9-item interview. Each item is rated from 0 (absent) to 3 (severe). The total CDSS score is calculated by summing the scores of all items [20,21]. A CDSS score greater than 6 was used to identify participants with significant depressive symptoms [22].

The BNSS is a semi-structured interview with 13 items and six subscales—one for each negative symptoms domain and an additional subscale for lack of normal distress. Each item is rated from 0 (normal) to 6 (extremely severe). Subscale scores are calculated by summing the item scores. The total BNSS score is calculated by summing all item scores [23,24,25,26]. SNS is a 20-item self-evaluation scale that assesses all five negative symptoms. A patient is asked to indicate whether they agree (2 points), neither agree nor disagree (1 point), or do not agree (0 points) with each statement. Subscale and total scores were calculated by summing the item scores [27,28,29]. A cutoff score of 7 or higher on the SNS was used to define clinically significant negative symptoms [30]. No cutoff scores have yet been established for the BNSS.

We calculated the prevalence of primary, secondary, prominent, and predominant negative symptoms using different criteria across psychometric scales. Negative symptoms were primary if there was a score of ≥4 on at least one item in the PANSS NS or a score of ≥3 on at least one BNSS item, and there were no PANSS PS scores of >19 or CDSS scores of >6. Negative symptoms were considered secondary if there were either clinically pronounced positive symptoms (PANSS PS scores of ≥19) or depressive symptoms (CDSS ≥ 6). Negative symptoms were prominent when there were at least three moderate (score ≥ 4) (PANSS4) or at least two moderately severe (score ≥ 5) (PANSS5) items on the PANSS NS [27]. We found no documented criteria for prominent and predominant negative symptoms in the BNSS and SNS, so we applied our own criteria, derived from the PANSS criteria reported in the literature. For the BNSS, the same rules applied as with PANSS—if there were at least three subscales with at least one item with a moderate score (≥3) (BNSS3) or two subscales with at least one item with a moderately severe score (≥4) (BNSS4), negative symptoms were prominent. Because SNS is graded differently compared to BNSS or PANSS, we used different criteria for it but kept the rule of at least three moderate negative symptoms from the PANSS criteria—negative symptoms were considered prominent if at least three subscales contained an item with a score ≥ 2. In all cases of PANSS NS, BNSS, and SNS, prominent negative symptoms were predominant if the PANSS PS was <19 and the CDSS TS was <6. Negative symptoms were clinically meaningful if the total score of SNS was ≥7, or of PANSS NS ≥ 20.

Health-related quality of life was assessed using the Short Form-36 (SF-36). The SF-36 is a multidimensional self-rated questionnaire with eight health domains (eight subscores): physical functioning (PF), role limitations due to physical health (RP), bodily pain (BP), general health (GH), vitality (VT), social functioning (SF), role limitations due to emotional problems (RE), and mental health (MH). Each of the eight domains is scored from 0 (worst) to 100 (best). Of particular interest to this study is the MH subscore of SF-36, which assesses mental health-related quality of life [31]. We chose the SF-36 over other QoL measures because it is widely used in the literature and is one of the few measures available in Lithuanian.

Statistical analyses were conducted in SPSS version 31. We used descriptive statistics to summarize demographic data and the prevalence of negative and depressive symptoms. The Shapiro–Wilk test was used to assess normality. Correlation analyses were used to assess relationships among variables. Pearson correlation was used for normally distributed variables, and Spearman’s rank correlation for non-normally distributed variables. A Bonferroni correction was applied after the correlation analysis. We used Student’s *t*-test for normally distributed variables or the Mann–Whitney U test for non-normally distributed variables to assess differences in SF-36 scores across patient groups. Linear regression was performed to identify which symptoms and sociodemographic variables were most independently associated with the SF-36 MH subscale score, after accounting for the contributions of other variables. This analysis was not designed to establish causal direction. Assumptions for linear regression, including the Variance Inflation Factor (VIF), Durbin–Watson (DW), standard residuals, Cook’s distance, and normality of the dependent variable, were assessed using a P-P plot and the Shapiro–Wilk statistic. Homoscedasticity was assessed using a scatterplot.

## 3. Results

### 3.1. Sociodemographic Data

A total of 323 participants were included in this study. The sample was nearly equally distributed by sex: 50.5% (n = 163) were male, and 49.5% (n = 160) were female. Sociodemographic data are detailed in Table 1. Most patients (85.1%, n = 275) had previously been hospitalized on a psychiatric inpatient ward. Most participants reported never having attempted suicide (57.3%, n = 185). The mean subjective general well-being score was 6.6 (SD 2.99, 95% CI 6.27–6.93). None of the demographic variables were normally distributed according to the Shapiro–Wilk test, and none differed between sexes (*p* > 0.05). Most of our patients had a diagnosis of schizophrenia (80%, n = 258). Almost a tenth (9.4% n = 30) had a diagnosis of schizoaffective disorder.

### 3.2. Clinical Symptom Scores

The mean PANSS NS score was 25.33 (SD = 6.86; 95% CI 24.57–26.09). The mean PANSS PS score was 20.9 (SD = 6.68; 95% CI 20.17–21.64). The mean total BNSS score was 36.48 (SD = 15.93; 95% CI 34.74–38.23). The mean total SNS score was 18.9 (SD = 8.99; 95% CI 17.91–19.89). Most patients had clinically relevant negative symptoms: 92.9% (n = 300) of the sample had an SNS score ≥ 7, and 80.2% (n = 259) had a PANSS-NS score ≥ 20.

Primary negative symptoms were less prevalent than secondary negative symptoms: PANSS NS group (19.5%, n = 63) and BNSS group (17%, n = 55). Most participants had prominent negative symptoms. The BNSS3 (68.1%, n = 220) and PANSS4 (71.8%, n = 232) prominent symptom groups showed similar prevalence. However, the BNSS4 group had a higher prevalence of prominent negative symptoms (58.8%, n = 190) than the PANSS5 group (48.3%, n = 156). SNS had a prevalence of prominent negative symptoms (56.7%, n = 183), similar to that of the PANSS and BNSS groups. However, a much smaller percentage of the sample had predominant negative symptoms: BNSS3 (13%, n = 42), BNSS4 (11.1%, n = 36), PANSS4 (14.6%, n = 47), PANSS5 (9.3%, n = 30), or SNS (10.5%, n = 34). As a detailed description of the various types of negative symptoms is not the focus of this article, we invite you to consult our previous article on prevalence data [18].

Depressive symptoms were not markedly pronounced in this inpatient sample, with a mean CDSS score of 5.73 (SD, 5.03; 95% CI, [5.18; 6.28]). Only the first item (Depression) of the CDSS had a mean score above 1 (mean = 1.16, SD = 1.02, 95% CI [1.05; 1.27]). Most patients had depressive symptom scores below 6 (57.9%, n = 187).

### 3.3. Quality of Life Scores

The PF subscale (mean = 75.39; SD = 25.37, 95% CI 72.6–78.18) and MH (mean = 51.25; SD = 19.79, 95% CI 49.08–53.43) had the highest mean scores, indicating the highest QoL ratings for physical functioning and mental health, respectively. The lowest mean score was on the BP subscale (31.15; SD = 30.3; 95% CI 27.82–34.47), indicating lower QoL related to BP. All SF-36 mean scores are shown in Table 2.

### 3.4. Correlation Analysis

A total of 42 correlation analyses were performed to assess relationships between SF-36 subscores and sociodemographic factors. After applying the Bonferroni correction, the adjusted *p*-value was 0.0012. None of the sociodemographic factors were normally distributed; therefore, Spearman correlation was used. The results of the correlation analysis are shown in Table 3. Age had a weak inverse correlation with some SF-36 subscores: PF (r = −0.18, *p* = 0.001), GH (r = −0.18, *p* = 0.001), VT (r = −0.13, *p* = 0.02). Weight showed a weak inverse correlation only with PF (r = −0.18, *p* < 0.001). Subjective general well-being score had weak to moderate correlations with all SF-36 subscores: PF (r = 0.43, *p* < 0.001), RP (r = 0.33, *p* < 0.001), BP (r = −0.22, *p* < 0.001), GH (r = 0.44, *p* < 0.001), VT (r = 0.42, *p* < 0.001), SF (r = 0.33, *p* < 0.001), RE (r = 0.22, *p* < 0.001), MH (r = 0.46, *p* < 0.001).

After performing Mann–Whitney tests and Student’s *t*-tests, we found that males had significantly higher scores on RP (median 50 vs. 25, *p* = 0.02) and MH (mean 54.48 vs. 48.15, *p* = 0.002) and lower scores on BP (median 22 vs. 33, *p* = 0.004) compared to females. Participants who had the first episode of psychosis had higher scores on PF (median 95 vs. 80, *p* < 0.001), RP (median 40 vs. 25, *p* < 0.001), GH (median 60 vs. 45, *p* < 0.001), SF (median 56 vs. 44, *p* = 0.02), and MH (mean 57.5 vs. 50, *p* = 0.02) compared to those without a first episode of psychosis. Participants who had a past suicide attempt had lower scores on PF (median 80 vs. 85, *p* < 0.001), BP (median 22 vs. 33, *p* = 0.012), GH (median 40 vs. 55, *p* < 0.001), VT (median 35 vs. 45, *p* = 0.01), SF (median 38.5 vs. 56, *p* < 0.001), and MH (mean 45.19 vs. 55.94, *p* < 0.001) compared to other participants. 

CDSS-TS was not normally distributed; therefore, Spearman’s rank correlation was used. Almost all SF-36 subscales correlated significantly with CDSS-TS: PF (r = −0.355, *p* < 0.001), RP (r = −0.234, *p* < 0.001), BP (r = 0.097, *p* = 0.082), GH (r = −0.42, *p* < 0.001), VT (r = −0.428, *p* < 0.001), SF (r = −0.351, *p* < 0.001), RE (r = −0.282, *p* < 0.001), MH (r = −0.554, *p* < 0.001).

A total of 32 correlation analyses were conducted to evaluate relationships between the eight SF-36 subscores and SNS TS, BNSS TS, PANSS NS, and MARDER NF. The adjusted *p*-value was 0.0016. Only PANSS NS was normally distributed. Therefore, the correlation between PANSS NS and SF-36 was Pearson, and all other correlations were Spearman’s. Correlations between negative symptom scores and SF-36 subscores are shown in Table 4. SNS TS correlated significantly with all SF-36 subscales. The strongest negative correlations were observed between SNS TS and VT (r = −0.55, *p* < 0.001) and MH (r = −0.47, *p* < 0.001). PANSS NS did not correlate significantly with SF-36 subscores. MARDER NF correlated significantly with PF (r = −0.224, *p* < 0.001), GH (r = −0.178, *p* < 0.001), and SF (r = −0.157, *p* = 0.01). BNSS TS correlated significantly with all SF-36 subscores except BP and RE; these correlations remained weak, ranging from 0.2 to 0.3.

Positive symptoms showed only weak (r < 0.2) or nonsignificant correlations with SF-36 subscores. No PANSS PS correlated significantly with SF-36 after Bonferroni correction. 

Mann–Whitney tests and Student’s *t*-tests did not show differences in mean SF-36 subscores between primary and secondary negative symptom groups using either PANSS or BNSS. Participants in the PANSS4 prominent negative symptoms group had lower mean PF (median 90 vs. 80, *p* = 0.006), GH (median 55 vs. 45, *p* = 0.03), VT (median 45 vs. 40, *p* = 0.004), and SF (median 56 vs. 44, *p* = 0.010) scores. Participants in the PANSS5 prominent negative symptoms group had lower PF (median 85 vs. 80, *p* < 0.001), VT (median 40 vs. 35, *p* = 0.02), and MH (mean 43.95 vs. 38, *p* = 0.01). No other prominent symptom groupings showed significant differences in SF-36 scores. Patients with predominant negative symptoms according to BNSS3 criteria had lower mean RE score (median 33 vs. 10, *p* = 0.008), and patients with predominant negative symptoms according to BNSS4 criteria had lower SF (median 44 vs. 40, *p* = 0.02) and RE (median 33 vs. 10, *p* = 0.003) scores. No other predominant symptom grouping had significant differences in SF-36 scores.

### 3.5. Regression Analysis

A linear regression analysis was conducted to assess the independent associations of sociodemographic factors (sex, age, weight, prior hospitalizations, history of suicide attempts, and the subjective well-being score), depressive, and negative symptom scales on the SF-36 MH subscale. Descriptive statistics for the linear regression predictor variables are presented in Table 5.

**Figure 1:**
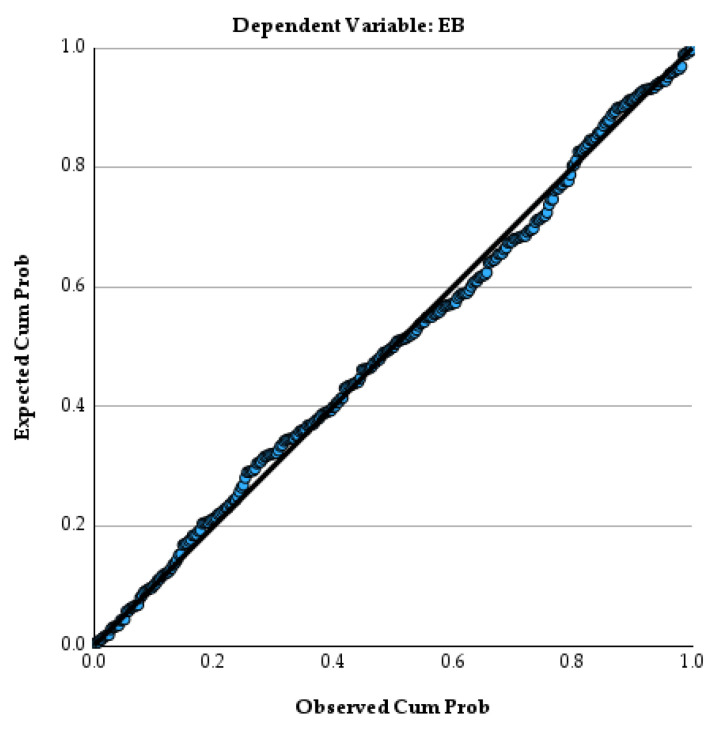
Normal P-P Plot of Regression Standardized Residual. Points close to the diagonal line indicate approximate normality of residuals.

**Figure 2:**
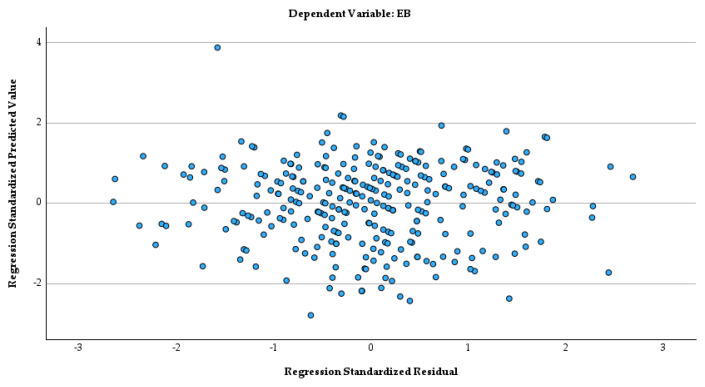
Standardized residuals against standardized predicted values scatterplot. Standardized residuals plotted against standardized predicted values; the absence of systematic patterns indicates homoscedasticity.

The regression model met the necessary assumptions. All VIF values were below 5, indicating no significant multicollinearity among predictors. The DW = 2.257 indicated no significant autocorrelation among residuals. No influential outliers were detected (all Cook‘s distances < 1, residual values < ±3). The normality of residuals was assessed using a P-P plot (Figure 1) and the Shapiro–Wilk test, which indicated a significant deviation from normality (*p* = 0.035). However, given the large sample size (n = 323), the Central Limit Theorem was assumed to hold, and the regression analysis was deemed appropriate [32,33]. The scatterplot (Figure 2) showed a random distribution of residuals, indicating homoscedasticity.

The overall regression model was statistically significant (F(10, 311) = 26.311, *p* < 0.001), accounting for 44% of the variance in SF-MH (R^2^ = 0.477, adjusted R^2^ = 0.441). Among the predictors, sex (B = −4.74, β = −0.12, *p* = 0.01), previous suicide attempt (B = −4.28, β = 0.11, *p* = 0.02), subjective well-being score (B = 1.07, β = 0.16, *p* < 0.001), CDSS TS (B = −1.43, β = −0.36, *p* < 0.001), and SNS TS (B = −0.72, β = −0.32, *p* < 0.001) were the varibales with statistically significant independent associations with SF-36 MH, indicating that higher scores on these scales are associated with lower mental health-related quality of life ratings. In contrast, MARDER NF (B = 0.003, β = 0.001, *p* = 0.99), BNSS TS (B = 0.13, β = 0.08, *p* = 0.13), previous hospitalizations (B = −0.71, β = −0.01, *p* = 0.29), weight (B = 0.08, β = 0.05, *p* = 0.09), and age (B = 0.09, β = 0.07, *p* = 0.19) were not statistically significant predictors in this model.

## 4. Discussion

We found that depressive symptoms of schizophrenia had the strongest correlation and showed the strongest independent association with QoL in multivariable analysis. Our results are similar to previous reports, indicating that QoL correlates more strongly with depressive symptoms than with negative symptoms [34]. Consistent with findings from Law et al., we observed that among various clinical parameters, the severity of depressive symptoms showed the strongest correlation with QoL [35]. Furthermore, Tan et al. found that depressive symptoms were more strongly correlated with QoL than any other psychopathological symptom group in patients with schizophrenia [8]. It is important to note that even though almost a tenth of our sample had a diagnosis of schizoaffective disorder, which might inflate the influence of depressive symptoms, less than half of the patients had a significant depressive symptom score, and the mean depressive symptom score was lower than 6, which is considered the lowest clinically meaningful score. These findings suggest that depressive symptoms may be an important target for improving QoL in schizophrenia. However, given the cross-sectional design, the directionality of this relationship cannot be established. Longitudinal and interventional studies are needed to determine whether treating depressive symptoms improves QoL.

Our model’s explained variance (44%) is comparable to that of Suttajit S et al., who found that negative symptoms, depression, and infrequent contact with relatives were significant predictors of overall quality of life in schizophrenia, explaining 47.2% [36]. However, unlike Suttajit S et al., who found that negative symptoms were the main predictor across all quality-of-life domains, we found that depressive symptoms had the strongest association with QoL. Similarly, Desalegn D et al. found that depressive and negative symptoms were inversely associated with psychological quality of life, with depressive symptoms often being the strongest predictor [11]. These associations highlight the potential value of systematic depression screening in schizophrenia care and warrant investigation in prospective interventional designs.

Overall, we found that the severity of negative symptoms was associated with lower QoL scores, and negative symptoms assessed with a second-generation scale showed a stronger correlation with QoL. Our results replicate findings from a meta-analysis by He et al., which found that more severe negative symptoms, as measured by the PANSS, were associated with worse QoL [3]. Consistent with our findings, Desalegn et al. found that negative symptoms were more strongly inversely associated with QoL than positive symptoms [9]. While there is limited direct comparative data on how BNSS versus PANSS negative symptom scores correlate with OoL, the BNSS’s broader coverage of negative symptom domains—especially motivation and pleasure—suggests it may better capture the aspects of negative symptoms that most impact subjective quality of life [24,37,38]. Therefore, our findings further support the association between more severe negative symptoms and worse QoL and add to the growing body of research showing that BNSS scores correlate more strongly with quality-of-life scores than negative PANSS scores do.

Negative symptoms assessed with a self-evaluation rating scale (SNS) showed stronger, more significant correlations with various SF-36 subscores than those assessed with observer-rated scales (BNSS, PANSS). Currently, there is an ongoing debate about whether self-reported or observer-rated psychopathology scores better reflect QoL. A consensus is emerging that observer-rated and self-rated scores reflect different aspects of functioning: observer-rated scales tend to reflect objective functioning more, whereas self-rated scales offer greater insight into the patient’s suffering [39,40]. The validity of SNS for predicting QoL has been demonstrated in previous research [41]. A recent review of self-rating scales for negative symptoms found that SNS was one of only two scales to receive a Grade A recommendation for use in clinical or research settings. SNS is designed to capture patients’ internal experiences and suffering, which are essential to understanding a person’s functioning, but cannot be observed or rated by an observer [42]. This systematic review supports the idea that negative symptoms measured with self-rated scales are more strongly correlated with subjective QoL than those measured with the BNSS, and our findings reinforce this.

Patients across the negative symptom groups did not show many significant differences in their QoL scores. Although some prior research suggested that primary negative symptoms were associated with poorer QoL, we did not replicate these findings. Patients with secondary negative symptoms likely also had pronounced depressive symptoms, which showed stronger associations with QoL. For example, a network analysis performed by Demyttenaere et al. found that even in a predominant negative symptoms group, depression and anxiety symptoms remained central [43]. Furthermore, consistent with findings from Rabinowitz et al., patients with prominent negative symptoms who exhibited at least three moderate negative symptoms had significantly lower QoL scores in some domains of physical functioning and mental health [44]. Patients with predominant negative symptoms, as measured by the BNSS, had lower QoL scores in the social functioning domain. Our results are consistent with those of Harvey et al., who found that patients in the predominant negative symptoms group showed no significant differences in QoL scores for everyday activities and work functioning, but had significantly worse scores in interpersonal functioning than patients with non-predominant negative symptoms [45]. We believe this pattern may reflect the nature of negative symptoms being linked more to the apathetic symptomatology leading to worse social and physical functioning, whereas depressive symptoms lead more to psychological suffering. However, causal conclusions cannot be drawn because of the study’s cross-sectional design.

The role of demographic factors in QoL research among patients with schizophrenia remains debated, with many studies reporting mixed results. We found that sociodemographic factors such as sex, history of suicidal attempts, and previous hospitalizations were less strongly linked to quality of life in patients with schizophrenia than depressive and negative symptoms measured with the self-evaluation scale, but showed a similar level of correlation to those assessed with second-generation scales. The subjective well-being score (1–10 Likert scale) showed moderate correlations with QoL, comparable to those of depressive symptom scores. Consistent with our findings, Dong M et al. observed that older age, female sex, and a history of hospitalization were significantly associated with QoL [4]. Other studies also replicate findings that female patients report lower QoL, consistent with ours [15,36]. Additionally, consistent with prior research, we found that prior suicidal attempts and a history of hospitalizations were linked to lower QoL [46,47].

The main limitations of our study were the lack of control for pharmacological treatment or illness duration, and the naturalistic, heterogeneous nature of our sample. This made it harder to distinguish among different types of negative symptoms and might have led to less refined results than those obtained in a tightly controlled sample. For example, unmeasured antipsychotic side effects, such as sedation or extrapyramidal symptoms, might confound QoL ratings and the prevalence of primary and predominant negative symptoms. Also, we obtained results from an inpatient sample, which limits generalizability to outpatient populations and may introduce selection bias. Moreover, heterogeneous negative symptom groupings might reduce statistical power to detect differences. The cross-sectional design precludes causal inference; the regression model identifies variables independently associated with QoL, not its determinants. Furthermore, we did not have a follow-up visit, which could have provided information about the persistence of negative and depressive symptoms and the influence of persistent symptoms on QoL. We believe this points to a direction for future research with multivariate analysis: dependent variable, a schizophrenia-specific QoL measure; independent variables: CDSS, SNS, BNSS, and a schizophrenia-specific cognitive functions measure; control variables (covariates): age, sex, chlorpromazine equivalent dose, presence or absence of anticholinergic drugs, side effect score (extrapyramidal, etc.), and length of hospital stay.

## 5. Conclusions

Our primary hypothesis that negative symptoms would show the strongest association with QoL was not confirmed; instead, depressive symptom severity showed the strongest and most consistent association with QoL across correlational and multivariable analyses, followed by self-rated negative symptoms. These findings are cross-sectional and hypothesis-generating. Future longitudinal studies should examine whether targeted treatment of depressive and self-rated negative symptoms improves QoL outcomes in schizophrenia.

## Figures and Tables

**Table 1:** Sociodemographic factors.

Variable	Male (Mean; SD)	Female (Mean; SD)	*p* Value
Age (years)	33.88 (10.98)	40.22 (14.32)	0.001
Weight (kg)	84.81 (18.99)	76.32 (19.71)	<0.001
Years of education	13.46 (2.47)	14.12 (2.98)	0.008
Work capacity (%)	65.51 (30.69)	65.40 (30.67)	0.661
Years of illness	11.87 (9.99)	15.45 (12.79)	0.006
Subjective well-being	6.45 (2.12)	6.54 (3.83)	0.766

SD—standard deviation; *p* value—significance level.

**Table 2:** Mean SF-36 scores.

SF-36 Subscales	Mean	SD	95% CI
PF	75.39	25.37	72.6–78.18
RP	42.29	40.18	37.88–46.7
BP	31.15	30.3	27.82–34.47
GH	47.88	25.52	45.3–50.46
VT	40.97	21.17	38.64–43.29
SF	47.87	26.73	44.93–50.8
RE	35.4	41.65	30.82–39.97
MH	51.25	19.79	49.08–53.43

PF—Physical Functioning subscale; RP—Role Physical subscale; BP—Bodily Pain subscale; GH—General Health subscale; VT—Vitality subscale; SF—Social Functioning subscale; RE—Role Emotional subscale; MH—Mental Health subscale.

**Table 3:** Spearman correlation analysis of SF-36 subscores and sociodemographic factors.

SF-36 Subscales	Age	Weight	YOE	WC	YOI	SWBS
PF	−0.129 *	−0.107	0.067	0.132 *	−0.131 *	0.43 **
RP	0.172 *	−0.024	−0.003	0.121 *	−0.129 *	0.326 **
BP	0.106	0.085	0.067	−0.053	0.15 *	−0.223 **
GH	−0.172 *	−0.092	0.11 *	0.186 **	−0.201 **	0.443 **
VT	−0.158 *	0.002	0.078	0.239 **	−0.145 *	0.416 **
SF	−0.015	−0.007	0.046	0.066	−0.051	0.332 **
RE	−0.048	0.022	−0.011	0.056	−0.051	0.224 **
MH	−0.001	0.046	0.053	0.146 *	−0.112 *	0.462 **

* *p* < 0.05, ** *p* < 0.0012. YOE—years of education; WC—work capacity; YOI—years of illness; SWBS—subjective well-being score; PF—Physical Functioning subscale; RP—Role Physical subscale; BP—Bodily Pain subscale; GH—General Health subscale; VT—Vitality subscale; SF—Social Functioning subscale; RE—Role Emotional subscale; MH—Mental Health subscale.

**Table 4:** Pearson and Spearman correlation of negative symptoms scores and SF-36.

SF-36 Subscales	SNS-TS	BNSS-TS	PANSS NS	MARDER NF
PF	−0.406 **	−0.259 **	−0.179 **	−0.224 **
RP	−0.23 **	−0.169 **	−0.113 *	−0.114 *
BP	0.119	0.090	0.010	0.038
GH	−0.409 **	−0.221 **	−0.111 *	−0.178 **
VT	−0.547 **	−0.221 **	−0.112 *	−0.135 *
SF	−0.399 **	−0.202 **	−0.118 *	−0.157 *
RE	−0.244 **	−0.043	0.004	−0.024
MH	−0.481 **	−0.129 *	−0.066	−0.101

* *p* < 0.05, ** *p* < 0.0012. SNS-TS—Self-evaluation of negative symptoms scale total score; BNSS-TS—Brief Negative Symptoms Scale total score; PANSS-NS = Positive and negative symptoms scale negative symptoms subscore; MARDER NF—Marder negative factor; PF—Physical Functioning subscale; RP—Role Physical subscale; BP—Bodily Pain subscale; GH—General Health subscale; VT—Vitality subscale; SF—Social Functioning subscale; RE—Role Emotional subscale; MH—Mental Health subscale.

**Table 5:** Descriptive statistics and bivariate correlations of linear regression predictor variables.

Predictor	Mean (SD)	Correlation with MH	*p* Value
Sex	0.5 (0.5)	−0.16	0.004
Age	36.61 (13.11)	−0.01	0.99
Weight	79.41 (19.45)	0.05	0.41
Previous hospitalizations	0.85 (0.36)	−0.13	0.03
Previous suicide attempts	0.57 (0.5)	0.27	<0.001
Subjective well-being score	6.6 (2.99)	0.45	<0.001
CDSS TS	5.73 (5.03)	−0.56	<0.001
SNS TS	18.9 (8.98)	−0.47	<0.001
MARDER NF	25.65 (7.95)	−0.13	0.02
BNSS TS	36.39 (15.88)	−0.14	0.01

SD—standard deviation; MH—SF-36 mental health subscore; *p* value—significance level; CDSS TS—total score of CDSS; SNS TS—total score of SNS; MARDER NF—Marder negative factor; BNSS TS—total score of BNSS.

## Data Availability

No supplementary data are available.

## References

[1] GBD 2019 Mental Disorders Collaborators (2022). Global, regional, and national burden of 12 mental disorders in 204 countries and territories, 1990–2019: A systematic analysis for the Global Burden of Disease Study 2019. Lancet Psychiatry.

[2] Wang Y.-C., Fan H.-Y., Huang Q.-H., Liu X.-C., Huang Y.-R., Su Z., Cheung T., Ungvari G.S., Feng Y., Wang G. (2025). Quality of life in patients with schizophrenia: A systematic review and meta-analysis of case-control studies. Schizophr. Res..

[3] He X.Y., Migliorini C., Huang Z.H., Wang F., Zhou R., Chen Z.L., Xiao Y.-N., Wang Q.-W., Wang S.-B., Harvey C. (2022). Quality of life in patients with schizophrenia: A 2-year cohort study in primary mental health care in rural China. Front. Public Health.

[4] Dong M., Lu L., Zhang L., Zhang Y.-S., Ng C.H., Ungvari G.S., Li G., Meng X., Wang G., Xiang Y.-T. (2019). Quality of Life in Schizophrenia: A Meta-Analysis of Comparative Studies. Psychiatr. Q..

[5] Rhee T.G., Rosenheck R.A. (2019). Does improvement in symptoms and quality of life in chronic schizophrenia reduce family caregiver Burden?. Psychiatry Res..

[6] Tolbert D.V., Mccollister K.E., Leblanc W.G., Lee D.J., Fleming L.E., Muennig P. (2014). The economic burden of disease by industry: Differences in quality-adjusted life years and associated costs. Am. J. Ind. Med..

[7] Zhou T., Hu H., Gao J., Yu H., Jit M., Wang P. (2024). Health-Related Quality of Life and Economic Burden Among Hospitalized Children with Hand, Foot, and Mouth Disease: A Multiregional Study in China. PharmacoEconomics Open.

[8] Tan X.W., Seow E., Abdin E., Verma S., Sim K., Chong S.A., Subramaniam M. (2019). Subjective quality of life among patients with schizophrenia spectrum disorder and patients with major depressive disorder. BMC Psychiatry.

[9] Desalegn D., Girma S., Abdeta T. (2020). Quality of life and its association with psychiatric symptoms and socio-demographic characteristics among people with schizophrenia: A hospital-based cross-sectional study. PLoS ONE.

[10] Rabinowitz J., Werbeloff N., Caers I., Mandel F.S., Stauffer V., Menard F., Kinon B.J., Kapur S. (2013). Negative symptoms in schizophrenia—The remarkable impact of inclusion definitions in clinical trials and their consequences. Schizophr. Res..

[11] Galderisi S., Kaiser S., Bitter I., Nordentoft M., Mucci A., Sabé M., Giordano G.M., Nielsen M.Ø., Glenthøj L.B., Pezzella P. (2021). EPA guidance on treatment of negative symptoms in schizophrenia. Eur. Psychiatry.

[12] European Medicines Agency (2012). Guideline on Clinical Investigation of Medicinal Products, Including Depot Preparations in the Treatment of Schizophrenia [Internet]. London. http://www.ema.europa.eu.

[13] Laughren T., Levin R. (2011). Food and drug administration commentary on methodological issues in negative symptom trials. Schizophr. Bull..

[14] Defar S., Abraham Y., Reta Y., Deribe B., Jisso M., Yeheyis T., Kebede K.M., Beyene B., Ayalew M. (2023). Health related quality of life among people with mental illness: The role of socio-clinical characteristics and level of functional disability. Front. Public Health.

[15] Rotstein A., Shadmi E., Roe D., Gelkopf M., Levine S.Z. (2022). Gender differences in quality of life and the course of schizophrenia: National study. BJPsych Open.

[16] Montvidas J., Kačerginis E., Petraitytė P., Zauka E., Adomaitienė V. (2025). Prevalence of Primary, Prominent, and Predominant Negative Symptoms of Schizophrenia in Routine Inpatient Psychiatric Care. Medicina.

[17] Kay S.R., Flszbeln A., Qpjer L.A. (1967). The Positive and Negative Syndrome Scale (PANSS) for Schizophrenia [Internet]. Volume 13. https://academic.oup.com/schizophreniabulletin/article/13/2/261/1919795.

[18] Marder S.R., Kirkpatrick B. (2014). Defining and measuring negative symptoms of schizophrenia in clinical trials. Eur. Neuropsychopharmacol..

[19] Marder S.R., Davis J.M., Chouinard G. (1997). The Effects of Risperidone on the Five Dimensions of Schizophrenia Derived by Factor Analysis. J. Clin. Psychiatry.

[20] Addington J., Shah H., Liu L., Addington D. (2014). Reliability and validity of the Calgary Depression Scale for Schizophrenia (CDSS) in youth at clinical high risk for psychosis. Schizophr. Res..

[21] Porter L., Jones C., Fox A. (2022). Reliability of the Calgary depression scale for schizophrenia: A meta-analysis. Schizophr. Res..

[22] Müller M.J., Brening H., Gensch C., Klinga J., Kienzle B., Müller K.M. (2005). The Calgary Depression Rating Scale for schizophrenia in a healthy control group: Psychometric properties and reference values. J. Affect. Disord..

[23] Kirkpatrick B., Strauss G.P., Nguyen L., Fischer B.A., Daniel D.G., Cienfuegos A., Marder S.R. (2011). The brief negative symptom scale: Psychometric properties. Schizophr. Bull..

[24] Tatsumi K., Kirkpatrick B., Strauss G.P., Opler M. (2020). The brief negative symptom scale in translation: A review of psychometric properties and beyond. Eur. Neuropsychopharmacol..

[25] Mucci A., Galderisi S., Merlotti E., Rossi A., Rocca P., Bucci P., Piegari G., Chieffi M., Vignapiano A., Maj M. (2015). The Brief Negative Symptom Scale (BNSS): Independent validation in a large sample of Italian patients with schizophrenia. Eur. Psychiatry.

[26] Montvidas J., Zauka E., Dollfus S., Kirkpatrick B., Adomaitienė V. (2025). Validation of the Lithuanian version of the brief Negative Symptoms Scale. Nord. J. Psychiatry.

[27] Dollfus S., Mach C., Morello R. (2016). Self-evaluation of negative symptoms. Schizophr. Bull..

[28] Montvidas J., Adomaitienė V., Leskauskas D., Dollfus S. (2021). Validation of the lithuanian version of the self-evaluation of negative symptoms scale (SNS). Nord. J. Psychiatry.

[29] Dollfus S., Mucci A., Giordano G.M., Bitter I., Austin S.F., Delouche C., Erfurth A., Fleischhacker W.W., Movina L., Glenthøj B. (2022). European Validation of the Self-Evaluation of Negative Symptoms (SNS): A Large Multinational and Multicenter Study. Front. Psychiatry.

[30] Dollfus S., Delouche C., Hervochon C., Mach C., Bourgeois V., Rotharmel M., Tréhout M., Vandevelde A., Guillin O., Morello R. (2019). Specificity and sensitivity of the Self-assessment of Negative Symptoms (SNS) in patients with schizophrenia. Schizophr. Res..

[31] Leese M., Schene A., Koeter M., Meijer K., Bindman J., Mazzi M., Puschner B., Burti L., Becker T., Moreno M. (2008). SF-36 scales, and simple sums of scales, were reliable quality-of-life summaries for patients with schizophrenia. J. Clin. Epidemiol..

[32] Royston J.P. (1982). An Extension of Shapiro and Wilk’s W Test for Normality to Large Samples. Appl. Statist [Internet]. https://academic.oup.com/jrsssc/article/31/2/115/6985178.

[33] Midway S., White J.W. (2025). Testing for normality in regression models: Mistakes abound (but may not matter). R. Soc. Open Sci..

[34] Narvaez J.M., Twamley E.W., McKibbin C.L., Heaton R.K., Patterson T.L. (2008). Subjective and objective quality of life in schizophrenia. Schizophr. Res..

[35] Law C.W., Chen E.Y.H., Cheung E.F.C., Chan R.C.K., Wong J.G.W.S., Lam C.L.K., Leung K.F., Lo M.S.M. (2005). Impact of untreated psychosis on quality of life in patients with first-episode schizophrenia. Qual. Life Res..

[36] Suttajit S., Pilakanta S. (2015). Predictors of quality of life among individuals with schizophrenia. Neuropsychiatr. Dis. Treat..

[37] Weigel L., Wehr S., Galderisi S., Mucci A., Davis J., Giordano G.M., Leucht S. (2023). The Brief negative Symptom Scale (BNSS): A systematic review of measurement properties. Schizophrenia.

[38] Mucci A., Vignapiano A., Bitter I., Austin S.F., Delouche C., Dollfus S., Erfurth A., Fleischhacker W.W., Giordano G.M., Gladyshev I. (2019). A large European, multicenter, multinational validation study of the Brief Negative Symptom Scale. Eur. Neuropsychopharmacol..

[39] Jung H.Y., Hwang S.S.H., Yi J.S., Kim Y., Kim Y.S. (2010). Clinician-rated functioning and patient-rated quality of life in schizophrenia: Implications of their correspondence for psychopathology and side effects. Prog. Neuropsychopharmacol. Biol. Psychiatry.

[40] Riedel M., Spellmann I., Schennach-Wolff R., Obermeier M., Musil R. (2011). The RSM-scale: A pilot study on a new specific scale for self- and observer-rated quality of life in patients with schizophrenia. Qual. Life Res..

[41] Montvidas J., Adomaitienė V., Leskauskas D., Dollfus S. (2023). Correlation of Health-Related Quality of Life with Negative Symptoms Assessed with the Self-Evaluation of Negative Symptoms Scale (SNS) and Cognitive Deficits in Schizophrenia: A Cross-Sectional Study in Routine Psychiatric Care. J. Clin. Med..

[42] Métivier L., Dollfus S. (2025). Systematic Review of Self-Assessment Scales for Negative Symptoms in Schizophrenia. Brain Sci..

[43] Demyttenaere K., Anthonis E., Acsai K., Correll C.U. (2022). Depressive Symptoms and PANSS Symptom Dimensions in Patients with Predominant Negative Symptom Schizophrenia: A Network Analysis. Front. Psychiatry.

[44] Rabinowitz J., Berardo C.G., Bugarski-Kirola D., Marder S. (2013). Association of prominent positive and prominent negative symptoms and functional health, well-being, healthcare-related quality of life and family burden: A CATIE analysis. Schizophr. Res..

[45] Harvey P.D., Davidson M., Saoud J.B., Kuchibhatla R., Moore R.C., Depp C.A., Pinkham A.E. (2024). Prevalence of prominent and predominant negative symptoms across different criteria for negative symptom severity and minimal positive symptoms: A comparison of different criteria. Schizophr. Res..

[46] Ponizovsky A.M., Grinshpoon A., Levav I., Ritsner M.S. (2003). Life Satisfaction and Suicidal Attempts among Persons with Schizophrenia. Compr. Psychiatry.

[47] Yan F., Xiang Y.-T., Hou Y.-Z., Ungvari G.S., Dixon L.B., Chan S.S.M., Lee E.H.M., Li W.-Y., Li W.-X., Zhu Y.-L. (2013). Suicide attempt and suicidal ideation and their associations with demographic and clinical correlates and quality of life in Chinese schizophrenia patients. Soc. Psychiatry Psychiatr. Epidemiol..

